# Ethical tightrope: Navigating neuro-ethics in brain computer interface (BCI) technology

**DOI:** 10.1016/j.bas.2024.102800

**Published:** 2024-04-01

**Authors:** Allah Yar Yahya Khan, Ammar Anjum, Haseeb Mehmood Qadri

**Affiliations:** Akhtar Saeed Trust Hospital, Lahore, Pakistan; Punjab Institute of Neurosciences, Lahore, Pakistan

Dear Editor,

With the advancements in the computer technology, the role of brain-computer interface (BCI) is becoming ever more promising in the field of medicine. We have already been familiar with BCI in the form of cochlear implants and auditory brain stem implants, used in patients with sensorineural hearing loss, but with modern advancements, the door to countless other possibilities has now been opened (Bergeron et al., 2023). Gadgets like brain-controlled prosthetics (BCPs) and brain-controlled speech synthesizers (BCSSs) are no longer science fiction, making these human aides promising for assisting individuals with disabilities. Not to mention its other myriads of utilities including, but not limited to: motor rehabilitation following trauma, deep brain stimulation for treating Parkinson's disease, studying the human emotional state and diagnosing various diseases for example schizophrenia, breast cancer and attention deficit (ADHD) to name a few (Mridha et al., 2021). These applications hold immense promise for shaping the future of medicine, potentially allowing transformative advancements in healthcare.

Although the potential benefits of BCI are undeniable, it is fundamental to critically examine the ethical implications of its implementation. Our objective is to shed light to these concerns and advocate for responsible development and deployment protocols of BCI technology. Ethical considerations of BCI encompass a range of issues including patient consent and agency. Many potential candidates of BCI suffer from various neurocognitive disorders, often comorbid with dementia, they may even develop symptoms during the course of the treatment thought initially asymptomatic, questioning full patient agency as patients are unable to provide.

The matters of obtaining paediatric consent also raises some ethical questions. Although legally children lack the capacity to make medical decision, their assent for BCI implantation is important, since BCI calibration requires extensive training sessions that would not be possible without the child's participation (Bergeron et al., 2023). Unwanted movements of robotic arm and wrong translation of patients thought by language BCI as a result of decoding defects can end up in embarrassing or dangerous situations.

The concerns regarding the target population are also worth noticing. The patient population benefitting the most from BCI typically do not make the most suitable research candidates. There are concerns that these patients with severe neurologic disabilities maybe choosing to participate in this study out of desperation, without considering all the risks associated with the interface.

The implantation of BCI sensor also raises the ethical concern regarding balancing the potential risks of the implant and its benefits, as studies have shown that 12% of the patients with brain implants experience serious adverse effects; hardware failure alone contribute to 50% one year failure rate of BCI **(**Davidoff et al., 2020). Some argue that it is unethical to put the patient in this unnecessary risk when other non-invasive treatment options exist. Despite the superiority of BCI compared to these options, the adverse effects associated with it should be adequately accounted for (Davidoff et al., 2020).

The representation of the users brain activity by the BCI should also be kept under notice, as BCI represents only a tiny fraction of the users brain function. The question of whether individuals should be held accountable for the actions performed using BCI arises due to its limitations in capturing the entire complexity of human cognition and decision-making process.

In conclusion, it is evident that while BCI technology holds immense promise, its ethical implications should not be overlooked. This can be achieved by ensuring adequate assessment of patients' decision-making capacity and careful choice of the target population. It is important that physicians weigh the drawbacks and benefits of BCI before its application, instead of jumping towards the "next best thing” as soon as it is available. It is important from an ethical perspective to realistically evaluate alternate treatment options as well and to adopt a patient-centered approach. We urge policy-makers, researchers and stake holders to prioritize neuro-ethics in the development and deployment of BCI. By fostering a framework that respects individual rights, promotes inclusivity and addresses societal concerns, we can harness the full potential of BCI while safeguarding against its potential pitfalls.

## Grant & financial disclosure

None.

## Ethical approval

I, Dr. Haseeb Mehmood Qadri, certify that this manuscript is a unique submission and is not being considered for publication, in part or in full, with any other source in any medium.

## Declaration of competing interest

The authors declare that they have no known competing financial interests or personal relationships that could have appeared to influence the work reported in this paper.
